# High‐Entropy Non‐Flammable Ionic Liquid/Dimethoxymethane Composite Electrolyte for High‐Performance Lithium‐Ion Batteries

**DOI:** 10.1002/advs.202417306

**Published:** 2025-03-17

**Authors:** Purna Chandra Rath, Chun‐Yen Chen, Jagabandhu Patra, Chun‐Chen Yang, Yu‐Sheng Su, Chien‐Te Hsieh, Wei‐Ren Liu, Ju Li, Jeng‐Kuei Chang

**Affiliations:** ^1^ Department of Materials Science and Engineering National Yang Ming Chiao Tung University 1001 University Road Hsinchu 30010 Taiwan; ^2^ Hierarchical Green‐Energy Materials (Hi‐GEM) Research Center National Cheng Kung University 1 University Road Tainan 70101 Taiwan; ^3^ Battery Research Centre of Green Energy and Department of Chemical Engineering Ming Chi University of Technology 84 Gongzhuan Road New Taipei City 243303 Taiwan; ^4^ International College of Semiconductor Technology National Yang Ming Chiao Tung University 1001 University Road Hsinchu 30010 Taiwan; ^5^ Department of Chemical Engineering and Materials Science Yuan Ze University 135 Yuandong Road Taoyuan 320315 Taiwan; ^6^ R&D Center for Membrane Technology 200 Chung Pei Road Chungli District Taoyuan 32023 Taiwan; ^7^ Department of Chemical Engineering Chung Yuan Christian University 200 Chung Pei Road Taoyuan 32023 Taiwan; ^8^ Department of Nuclear Science and Engineering and Department of Materials Science and Engineering Massachusetts Institute of Technology 77 Massachusetts Avenue Cambridge MA 02139 USA

**Keywords:** electrolyte engineering, graphite compatibility, high safety, high voltage, operando analysis, synergistic additive effects

## Abstract

The development of high‐energy‐density and high‐safety lithium‐ion batteries requires advancements in electrolytes. This study proposes a high‐entropy ionic liquid/ether composite electrolyte, which is composed of *N*‐propyl‐*N*‐methylpyrrolidinium bis(trifluoromethanesulfonyl)imide (PMP–TFSI) ionic liquid, dimethoxymethane (DME), lithium difluoro(oxalato)borate (LiDFOB), fluoroethylene carbonate (FEC), and 1,1,2,2‐tetrafluoroethyl‐2,2,3,3‐tetrafluoropropyl ether (TTE). In this electrolyte, a unique coordination structure forms, where Li^+^ is surrounded by a highly complex environment consisting of DME, FEC, TTE, TFSI^−^, DFOB^−^, and PMP^+^. The effects of this solution structure on the solid‐electrolyte interphase chemistry and Li^+^ desolvation kinetics are examined. The proposed electrolyte has low flammability, high thermal stability, negligible corrosivity toward an Al current collector, and the ability to withstand a high potential of up to 5 V. Importantly, this electrolyte is highly compatible with graphite and SiO*_x_* anodes, as well as a high‐nickel LiNi_0.8_Co_0.1_Mn_0.1_O_2_ cathode. Operando X‐ray diffraction data confirm that the co‐intercalation of DME and PMP^+^ into the graphite lattice, a long‐standing challenge, is eliminated with this electrolyte. A 4.5‐V LiNi_0.8_Co_0.1_Mn_0.1_O_2_//graphite full cell with the proposed high‐entropy electrolyte is shown to have superior specific capacity, rate capability, and cycling stability, demonstrating the great potential of the proposed electrolyte for practical applications.

## Graphical abstract

An ionic liquid/ether composite high‐entropy electrolyte with a unique Li^+^ coordination structure that leads to an upgraded solid‐electrolyte interphase and improved Li^+^ desolvation kinetics is developed. It exhibits high thermal stability, negligible corrosivity, and a large potential window, and is highly compatible with graphite, SiO*_x_*, and high‐nickel LiNi_0.8_Co_0.1_Mn_0.1_O_2_ electrodes, showing great potential for battery applications.

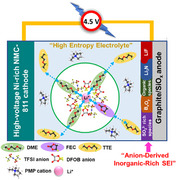

## 1 Introduction

Lithium‐ion batteries (LIBs) are crucial energy storage devices for a wide range of applications, such as portable electronics, electric vehicles, and grid‐scale energy storage systems, owing to their cost‐effectiveness, versatility, and longevity.^[^ ^1^, ^2^ ^]^ Higher energy density, greater reliability, and better safety are the main goals of LIB research and development.^[^ ^3^, ^4^ ^]^ However, the current LIB electrolyte is unable to meet increasing demands. For example, a high operation voltage is needed to extract a high capacity from cathodes (such as LiCoO_2_, LiNi_0.8_Co_0.1_Mn_0.1_O_2_ [NMC‐811]). However, the conventional carbonate electrolyte can barely withstand a potential beyond ≈4.3 V (vs Li^+^/Li) as it is limited by its anodic decomposition.^[^ ^5^, ^6^ ^]^ Moreover, the commonly used LiPF_6_ in the electrolyte is not an ideal Li salt because of its poor thermal stability and high moisture sensitivity.^[^ ^7^, ^8^ ^]^ Several highly reactive species, such as POF_3_, PF_5_, and HF, can be generated, leading to the corrosion of the active and inactive components (anodes, cathodes, current collectors, solid‐electrolyte interphase [SEI], and other battery parts) of LIBs and thus the deterioration of charge‐discharge performance.^[^ ^5^, ^9^ ^]^ Moreover, the conventional electrolyte is unstable, highly volatile, and flammable.^[^ ^10^, ^11^ ^]^ The development of high‐voltage electrolytes with superior reliability and safety is urgently required. It is noted that tuning the electrolyte composition is easier and more effective for improving LIB performance than developing sophisticated electrode materials and architectures.^[^ ^12^, ^13^ ^]^ The electrolyte is thus a critical component that determines the performance of LIBs.

Electrolytes with improved stability and non‐flammability for realizing high‐energy density and high‐safety LIBs have been actively pursued. Room‐temperature ionic liquids (ILs) are considered a potential electrolyte for advanced LIBs due to their low flammability, non‐volatility, wide electrochemical stability window, high thermal stability, and environmental friendliness.^[^ ^14^, ^15^, ^16^ ^]^ However, ILs usually have a high viscosity (difficult to wet separators and electrodes) and relatively low ionic conductivity.^[^ ^17^, ^18^ ^]^ Unsatisfactory electrode rate capability at room temperature has long been a challenge for IL electrolytes.^[^ ^19^, ^20^ ^]^ In addition, their relatively high cost is a concern.^[^ ^21^, ^22^ ^]^ To overcome these problems, some studies developed IL‐based composite electrolytes by incorporating a low‐viscosity carbonate solvent.^[^ ^23^, ^24^ ^]^ Compared to a carbonate solvent, an ether solvent, especially dimethoxyethane (DME), can be more effective for incorporation into ILs.^[^ ^25^, ^26^ ^]^ Notably, ethers have been shown to be promising in a variety of battery applications because of their excellent reductive stability, low melting point, low viscosity, and high ionic conductivity.^[^ ^27^, ^28^, ^29^, ^30^, ^31^ ^]^ However, the use of IL‐ether composite electrolytes for carbonaceous anodes has been rarely reported. DME with a high donor number has a co‐intercalation tendency (together with Li^+^) into graphite.^[^ ^32^, ^33^ ^]^ ILs with bis(trifluoromethanesulfonyl)imide (TFSI^−^) anions, which are a favorable type of IL electrolyte, exhibit cation co‐intercalation into the graphite lattice, leading to irreversible electrode degradation.^[^ ^34^, ^35^ ^]^ Therefore, there have been no successful applications of TFSI‐based IL/DME composite electrolytes for LIBs. A previous study adopted *N*‐propyl‐*N*‐methylpyrrolidinium bis(fluorosulfonyl)imide (PMP–FSI)/1,1,2,2‐Tetrafluoroethyl‐2,2,3,3‐tetrafluoropropyl ether (TTE) (1:1) to ensure reversible Li^+^ intercalation/deintercalation of a graphite anode.^[^ ^36^ ^]^ However, FSI^−^ is highly corrosive toward Al,^[^ ^37^ ^]^ so cathode compatibility is a problem. Further, FSI‐based ILs have relatively low thermal stability.^[^ ^38^ ^]^ Many studies overlook these limitations of FSI‐based IL electrolytes for practical LIB applications. In addition, TTE is relatively expensive and has clearly higher viscosity compared to that of DME.^[^ ^39^ ^]^ The present study will investigate the use of a TFSI‐based IL/DME electrolyte for high‐voltage and high‐reliability LIBs.

Although Li metal is considered to be the ultimate anode for Li batteries owing to its high theoretical capacity (3860 mAh g^−1^) and low electrochemical potential (−3.04 V vs standard hydrogen electrode),^[^ ^40^, ^41^ ^]^ the growth of Li dendrites and low charge‐discharge Coulombic efficiency (CE) lead to early cell death and serious safety concerns.^[^ ^42^, ^43^ ^]^ Thus, rechargeable Li‐metal batteries are not yet fully practical. Carbonaceous and Si‐based materials are still the dominant anodes for typical LIBs. Therefore, they are considered in the present study. To enhance the battery energy density, high‐nickel layer‐structure cathodes with a high operation voltage are adopted. It has been recently found that high surface reactivity induces cathode particle pulverization and battery degradation.^[^ ^44^, ^45^ ^]^ Electrolytes with low reactivity have been shown to reduce particle cracking and improve battery life.^[^ ^46^ ^]^ Proper electrolyte selection is more effective for suppressing the performance deterioration of a high‐nickel cathode at a high operation voltage compared to the complicated tailoring of synthesis methods and thus cathode microstructures to alleviate the particle strain generated during cycling.^[^ ^47^ ^]^ Therefore, the development of a non‐flammable IL/ether composite electrolyte in this study considers compatibility with a high‐nickel cathode.

The concept of high entropy, which has recently attracted increasing attention, offers a new path for the formulation of high‐performance electrolytes.^[^ ^48^, ^49^, ^50^ ^]^ High‐entropy electrolytes consist of multiple salts, solvents, and additives. A complex solution structure is expected to form due to the high diversity of local interactions between Li^+^ and various anions, solvents, and additives. Higher Li^+^ diffusion kinetics, a lower melting point, increased dissolution of Li salts, improved redox stability, and reduced Li^+^ desolvation energy of electrolytes due to high‐entropy effects, which increase the degree of disorder and reduce the Gibbs free energy of the electrolytes, have been reported.^[^ ^51^, ^52^ ^]^ However, research on high‐entropy electrolytes is still a nascent stage; the detailed mechanisms need further investigation. Of note, the high‐entropy effects on IL‐based electrolytes have rarely been examined. In this context, a novel non‐flammable PMP–TFSI IL/DME high‐entropy electrolyte is developed in this work. The functions of each electrolyte constituent are systematically examined.

This study proposes a high‐entropy electrolyte that is co‐intercalation free, stable at high voltage, and non‐flammable. First, the effects of Li salts (namely LiPF_6_, LiTFSI, LiFSI, and Li difluoro(oxalato)borate [LiDFOB]) are examined. Their influence on the anodic potential limits, associated with Al pitting corrosion, is evaluated. Then, fluoroethylene carbonate (FEC) and TTE additives are introduced to modify the properties of the PMP–TFSI/DME composite electrolyte. This is the first study to explore the functions of these dual additives in an IL electrolyte. Operando X‐ray diffraction (XRD) data confirm that the additives ensure reversible Li^+^ intercalation/deintercalation at the graphite anode without DME solvent and IL cation co‐intercalation. Moreover, synergistic interaction between FEC and TTE is demonstrated for the first time. This interaction optimizes the Li^+^ coordination structure in the electrolyte and alters the SEI composition, leading to superior charge–discharge performance (including reversible capacity, rate capability, and cyclability) of graphite and silicon‐based anodes. In addition, the proposed electrolyte is compatible with Ni‐rich NMC‐811, mitigating cathode surface reactivity and capacity decay upon cycling. The high‐entropy 1 m LiDFOB PMP–TFSI/DME electrolyte with FEC and TTE dual additives, which is thermally stable and non‐flammable, is used for a 4.5‐V NMC‐811//graphite full cell to validate its practical application.

## 2 Results and Discussion

Figure S1a (Supporting Information) shows the XRD pattern of NMC‐811 powder. All the diffraction peaks belong to a rhombohedral (R3 m) crystal structure (JCPDS card no. 90‐0063); there are no impurity phases. The particle morphology examined using scanning electron microscopy (SEM) is shown in Figure S1b (Supporting Information). The nearly spherical NMC‐811 particles consist of tightly packed submicrometer primary grains. This type of hierarchical structure is favorable for shortening the Li^+^ transport distance and achieving a high compaction density of the active material. Figure S1c (Supporting Information) shows a high‐resolution transmission electron microscopy (TEM) image, in which a highly ordered NMC‐811 lattice can be observed. The measured inter‐plane distances of 0.47 and 0.29 nm correspond to the (003) and (101) planes of NMC‐811, respectively. Figure S1d (Supporting Information) shows the XRD pattern of the graphite powder, which exhibits a strong (002) basal plane diffraction peak at 2θ = 26.6°, corresponding to a *d*‐spacing of 3.35 Å. The obtained pattern belongs to an ideal graphite crystal structure (JCPDS card no. 75–2078). The SEM image in Figure S1e (Supporting Information) indicates that the spherical graphite particles have an average diameter of ≈10 µm.

**Figure 1:**
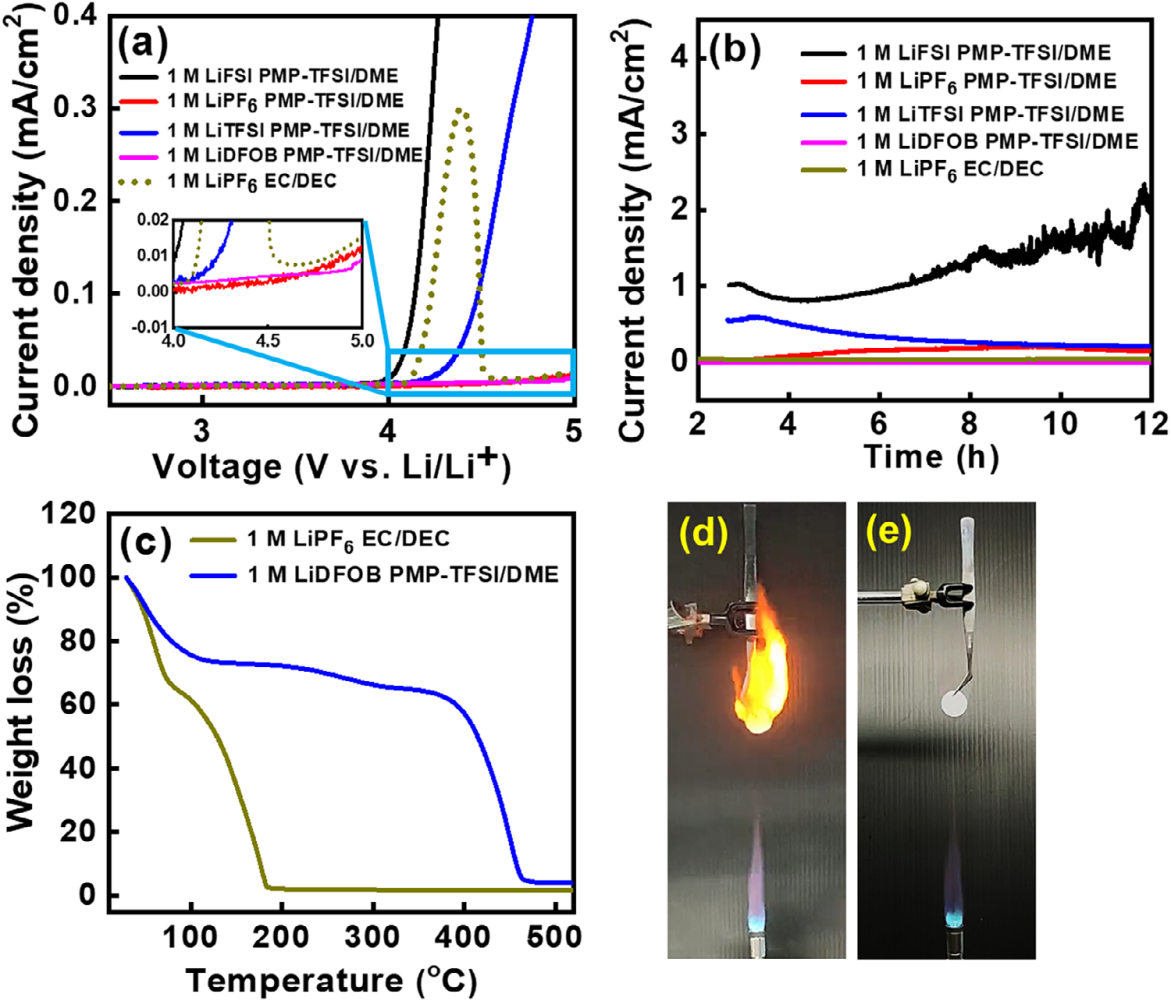
(a) Electrochemical stability windows of various electrolytes recorded at Al electrodes. (b) Chronoamperometry data of Al electrodes recorded at 5 V in various electrolytes. (c) TGA data and flammability tests of (d) 1 M LiPF_6_ EC/DEC and (e) 1 M LiDFOB PMP−TFSI/DME electrolytes.

The electrochemical stability windows of PMP−TFSI/DME (1:1 by volume) electrolytes with various Li salts (concentration: 1 M) at Al electrodes were examined; the obtained linear sweep voltammetry data are shown in **Figure** **1**a. The onset potentials of irreversible oxidation emerge at ≈4.0, ≈4.3, ≈4.9, and ≈5.0 V (vs Li^+^/Li) for LiFSI, LiTFSI, LiPF_6_, and LiDFOB electrolytes, respectively.^[^ ^53^, ^54^ ^]^ FSI^−^ and TFSI^−^ anions are corrosive toward Al current collectors (see Figure S2, Supporting Information), leading to limited stability windows. In contrast, PF_6−_ and DFOB^−^ anions can be anodically decomposed to form protective layers that suppress Al corrosion. The onset potential of ≈4.2 V for conventional 1 M LiPF_6_ ethylene carbonate (EC)/diethyl carbonate (DEC) (1:1 by volume) electrolyte is associated with organic carbonate solvent decomposition. Figure 1b shows the corresponding chronoamperometry data recorded at 5 V. After 12 h, the measured current densities in the LiFSI, LiTFSI, LiPF_6_, and LiDFOB electrolytes were 2095, 207, 140, and 0.5 µA cm^−2^, respectively. The large current fluctuation found with the LIFSI electrolyte is associated with the passivation breakdown and significant Al pitting corrosion (as supported by Figure S2a, Supporting Information).^[^ ^37^ ^]^ The LiDFOB electrolyte shows the best compatibility with the Al current collector. The side reaction current is lower than that for the conventional electrolyte (0.7 µA cm^−2^). Therefore, the PMP−TFSI/DME electrolyte with 1 M LiDFOB, denoted as PT/DME was used in subsequent electrochemical measurements. Figure 1c compares the thermogravimetric analysis (TGA) data of the conventional carbonate electrolyte and the proposed PT/DME electrolyte. The former electrolyte exhibits a significant weight loss of ≈40% before 100 °C, where the solvent violently evaporates and LiPF_6_ decomposes into LiF and PF_5_. At ≈185 °C, there was almost no residue on the TGA crucible. In contrast, the thermal stability of the latter electrolyte is clearly higher, with lower volatility and much less weight loss before ≈400 °C. In the TGA curve, multiple‐step weight loss can be observed, including the volatilization of DME below 100 °C, the decomposition of LiDFOB at ≈250 °C, and the breakdown of the PT IL at ≈400 °C. PMP−TFSI/DME electrolytes with other Li salts showed similar TGA curves; the data are presented in Figure S3 (Supporting Information). The flammability testing results in Figure 1d indicate that the conventional electrolyte ignites easily and burns violently, whereas the PMP−TFSI/DME electrolytes are non‐flammable (Figure 1e). The above results validate the high stability and high safety of the proposed IL‐ether composite electrolyte.

**Figure 2:**
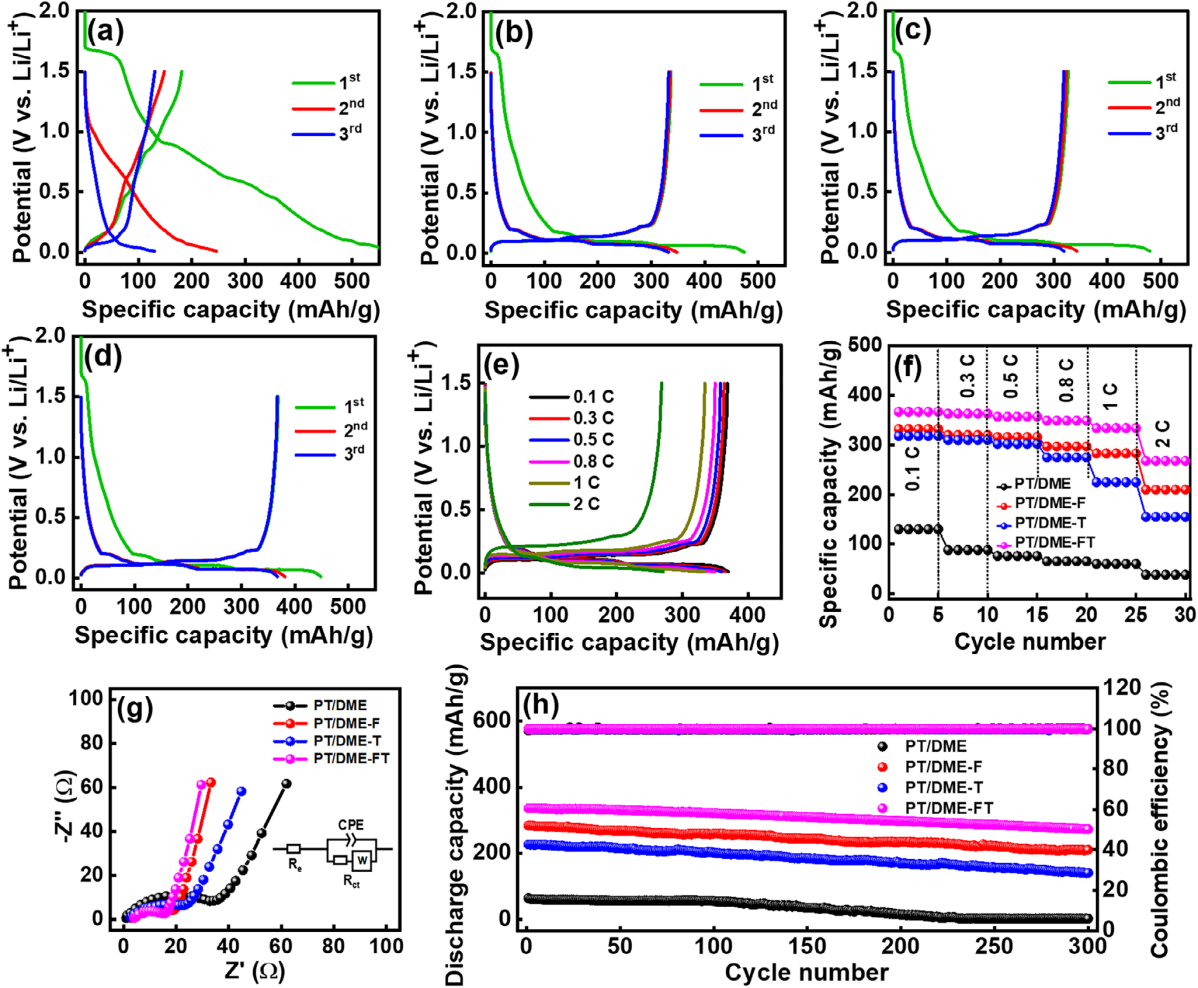
Initial charge‐discharge curves of graphite anodes measured at 0.1 C in (a) PT/DME, (b) PT/DME‐F, (c) PT/DME‐T, and (d) PT/DME‐FT electrolytes. (e) Charge‐discharge profiles of graphite anode measured at various rates in PT/DME‐FT electrolyte. (f) Comparative rate performance, (g) EIS spectra, and (h) cycling stability data (at 1 C) of graphite anodes with various electrolytes.

**Figure** **2**a shows the galvanostatic charge–discharge curves of a graphite half cell with the PT/DME electrolyte at 0.1 C (1 C = 372 mAh g^−1^). Highly polarized lithiation/delithiation curves with large irreversible capacity and low first‐cycle CE (i.e., 31%) can be observed. The decomposition products of the PT/DME electrolyte fail to form a stable SEI that can suppress the continuous breakdown of the electrolyte and co‐intercalation of DME and PMP^+^, which ultimately leads to graphite exfoliation and poor electrode reversibility.^[^ ^33^, ^34^ ^]^ To improve the graphite anode performance, FEC, TTE, or dual FEC/TTE additives were incorporated into the PT/DME electrolyte (denoted as PT/DME‐F, PT/DME‐T, and PT/DME‐FT, respectively). The viscosity and conductivity values of various electrolytes are shown in **Table** **1**. The PT/DME electrolyte shows much lower viscosity and higher conductivity compared to those of a plain PT IL electrolyte (e.g., 202.9 cP and 1.2 mS cm^−1^, respectively),^[^ ^23^ ^]^ indicating that the incorporation of DME is highly beneficial. The addition of FEC, TTE, or dual FEC/TTE additives can further reduce viscosity and improve conductivity. The PT/DME‐FT electrolyte shows viscosity and conductivity values of 8.2 cP and 6.3 mS cm^−1^, respectively, which are close to those of the conventional 1 M LiFP_6_ EC/DEC electrolyte (3.8 cP and 7.0 mS cm^−1^, respectively). The TGA and flammability data in Figure S4 (Supporting Information) indicate that the addition of FEC, TTE, or FEC/TTE does not significantly alter the thermal stability and safety of the composite electrolytes. Figure S5 (Supporting Information) shows that the electrochemical stability windows of the electrolytes with various additives are similar.

**Table 1:** The viscosity and ionic conductivity values of various electrolytes.

Electrolyte	Viscosity [cP]	Ionic conductivity [mS cm^−1^]
PT/DME	9.2	5.5
PT/DME‐F	8.5	6.0
PT/DME‐T	7.8	6.6
PT/DME‐FT	8.3	6.3

The initial three charge–discharge curves of graphite half cells with PT/DME‐F and PT/DME‐T electrolytes recorded at 0.1 C are respectively shown in Figure 2b,c. With the incorporation of FEC and TTE additives, the electrode reversibility clearly improved, and the charge–discharge plateaus below 0.2 V appeared. The best performance was obtained with the PT/DME‐FT electrolyte, as shown in Figure 2d. The first‐cycle CE values for the PT/DME‐F, PT/DME‐T, and PT/DME‐FT cells are 71, 68, and 82%, respectively. The increased CE (compared to 31% for the PT/DME cell) is attributed to the formation of an effective SEI, which suppressed DME and PMP^+^ co‐intercalation. The initial CE for PT/DME‐FT is similar to that for the conventional carbonate electrolyte (≈81%; see Figure S6, Supporting Information). This is the first study to demonstrate smooth Li^+^ intercalation and deintercalation at a graphite electrode in a TFSI‐based IL/ether composite electrolyte. The synergistic interaction between FEC and TTE additives is discussed later.

Figure 2e shows the charge–discharge profiles of the graphite anode measured at various rates in the PT/DME‐FT electrolyte after two conditioning cycles. The data for other cells are shown in Figure S7 (Supporting Information). The reversible capacities obtained at 0.1 C are 130, 332, 318, and 367 mAh g^−1^ for the PT/DME, PT/DME‐F, PT/DME‐T, and PT/DME‐FT cells, respectively. As shown in Figure 2f, the specific capacities of these cells decrease to 38, 210, 155, and 268 mAh g^−1^, respectively, at 2 C, corresponding to 29%, 63%, 49%, and 73% of the capacities obtained at 0.1 C. Figure 2g shows the electrochemical impedance spectroscopy (EIS) data of the graphite electrodes acquired in various electrolytes after two conditioning cycles. The Nyquist spectra are composed of a semicircle at high frequency and a sloping line at low frequency, which can be characterized by the equivalent circuit shown in the figure inset, where *R*_e_, *R*_ct_, *CPE*, and *W* are the electrolyte resistance, interfacial charge transfer resistance, constant‐phase element, and Warburg impedance associated with Li^+^ diffusion within the electrode, respectively.^[^ ^55^ ^]^ The *R*_ct_ values for the PT/DME, PT/DME‐F, PT/DME‐T, and PT/DME‐FT cells are 32.7, 17.5, 21.0, and 15.2 Ω, respectively. The apparent Li^+^ diffusion coefficient (*D*_Li_^+^) can be estimated from the sloping line in the low‐frequency region.^[^ ^56^ ^]^ The calculated *D*_Li_^+^ values for the four electrodes are 2.5, 3.4, 2.8, and 3.6 × 10^−10^ cm^2^ s^−1^, respectively. Both the *R*_ct_ and *D*_Li_^+^ data support the PT/DME‐FT cell having the best high‐rate performance among the cells studied. This is associated with the solution structures and SEI properties of the various electrolytes, as examined later. As shown in **Table** **2**, the maximum capacity and rate capability of the graphite cell with the PT/DME‐FT electrolyte are clearly better than those with the conventional 1 M LiPF_6_ EC/DEC electrolyte. Accordingly, the proposed electrolyte has great potential for practical applications.

**Table 2:** Summary of electrochemical performance of graphite anodes in various electrolytes.

	PT/DME	PT/DME‐F	PT/DME‐T	PT/DME‐FT	1 M LiPF_6_ EC/DEC
Initial CE	31%	71%	68%	82%	81%
Capacity (mAh g^−1^) @0.1 C	130	332	318	367	358
@0.3 C	88	320	310	363	353
@0.5 C	76	316	302	357	334
@0.8 C	65	297	277	349	304
@1 C	62	283	225	334	275
@2 C	38	210	155	268	198
High‐rate retention ^a)^	29%	63%	49%	73%	55%
Capacity retention after 300 cycles	1%	73%	61%	82%	72%

^a)^ Comparison between reversible capacities at 2 C and 0.1 C.

Figure 2h shows the cycling stability data of various cells measured at 1 C. After 300 charge‐discharge cycles, the graphite anodes retained 1, 73, 61, and 82% of their initial capacities in PT/DME, PT/DME‐F, PT/DME‐T, and PT/DME‐FT electrolytes, respectively. The fast capacity fading in the PT/DME electrolyte is due to the lack of an appropriate SEI and exfoliation of the graphite electrode.^[^ ^33^, ^34^ ^]^ Owing to the synergy of the dual additives, the best cyclability was obtained for the PT/DME‐FT cell. It is noted that the control cell with the conventional LiPF_6_ EC/DEC electrolyte retained 72% of its initial capacity after the same number of cycles (Table 2), confirming the advantage of the PT/DME‐FT electrolyte. The impedance evolution of the graphite electrodes upon cycling was also investigated (see Figure S8a, Supporting Information). As shown in Figure S8b (Supporting Information), the PT/DME cell exhibits the highest *R*_ct_ increase rate after 300 cycles, whereas the PT/DME‐FT cell shows a relatively stable *R*_ct_ upon cycling. The consistent trend between the data in Figure 2h and Figure S8b (Supporting Information) indicates that the graphite performance fading is mainly associated with interface deterioration. The morphologies of the graphite electrodes after cycling in PT/DME and PT/DME‐FT electrolytes shown in Figure S8c,d (Supporting Information) support this argument.

**Figure 3:**
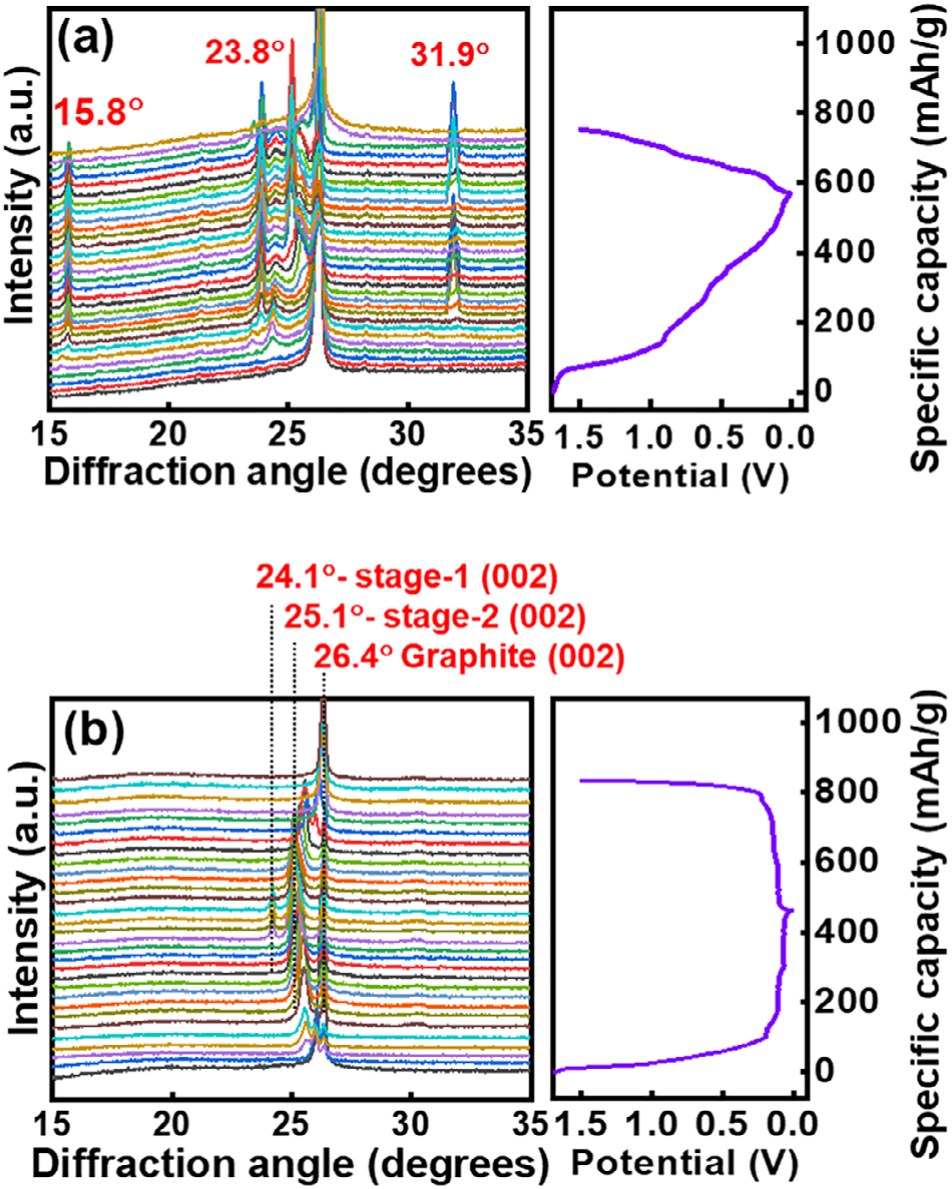
Operando XRD patterns and voltage profiles of graphite anodes measured in (a) PT/DME and (b) PT/DME‐FT electrolytes.

To examine the intercalation/deintercalation mechanism, operando XRD was employed to study the PT/DME and PT/DME‐FT cells. **Figure** **3** shows the obtained diffraction patterns along with the voltage profiles during electrode lithiation and delithiation reactions. Before charging (i.e., lithiation), the graphite (002) peak appears at 2θ = 26.6°, corresponding to an ideal interlayer distance of 3.35 Å.^[^ ^57^ ^]^ Upon Li^+^ insertion and desertion, while the graphite lattice expands and contracts, the (002) peaks could shift and new diffraction peaks may form.^[^ ^30^ ^]^ For the PT/DME cell (Figure 3a), in addition to the original graphite and graphite interaction peaks, a series of new peaks at 2θ values of 15.8°, 23.8°, 24.3°, and 31.9° appear at the late stage of charging. These peaks are associated with the co‐intercalation of DME molecules and PMP^+^ cations.^[^ ^33^, ^58^ ^]^ For the PT/DME‐FT cell (Figure 3b), at the beginning of lithiation, the graphite (002) peak gradually shifts to a lower diffraction angle of 25.1° due to the expansion of the graphite lattice. This corresponds to the formation of a stage‐2 compound (i.e., LiC_12_ phase) with a carbon interlayer distance of 3.54 Å.^[^ ^59^ ^]^ Further lithiation causes the emergence of a new peak at ≈24.01°, originating from a stage‐1 compound (i.e., LiC_6_ phase) with an interlayer distance of 3.70 Å.^[^ ^30^, ^59^ ^]^ No co‐intercalation peak was detected. During delithiation, excellent reversibility was observed since the graphite (002) peak was fully restored. To avoid solvent co‐intercalation, an EC‐based carbonate electrolyte is usually used for graphite anodes. The observed shift and splitting of the graphite (002) peak in the PT/DME‐FT electrolyte resembles the behavior of the EC‐based electrolyte.^[^ ^60^ ^]^ Even though the PT/DME‐FT composite electrolyte is EC‐free, it ensures greatly reversible Li^+^ intercalation/deintercalation reactions of the graphite anode.

**Table 3:** Summary of electrochemical performance of NMC‐811 cathodes in various electrolytes.

	PT/DME	PT/DME‐FT	1 M LiPF_6_ EC/DEC
Capacity (mAh g^−1^) @0.1 C	209	215	205
@0.3 C	185	200	182
@0.5 C	172	192	169
@0.8 C	165	184	160
@1 C	156	179	154
@2 C	147	172	145
High‐rate retention ^a)^	70%	80%	70%
Capacity retention after 200 cycles	57%	80%	50%

^a)^ Comparison between reversible capacities at 2 C and 0.1 C.

**Figure 4:**
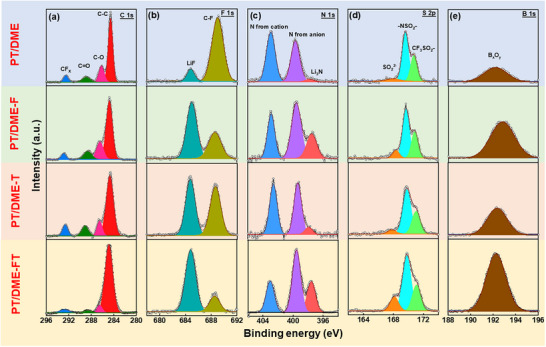
XPS (a) C 1s, (b) F 1s, (c) N 1s, (d) S 2p, and (e) B 1s spectra of graphite anodes acquired after 30 charge‐discharge cycles in various electrolytes.

X‐ray photoelectron spectroscopy analyses were performed for various graphite electrodes after 30 charge‐discharge cycles to gain insight into SEI chemistry. **Figure** **4**a shows the obtained C 1s spectra, which can be split into several constituents. In addition to the main C─C bonding peak at ≈284.6 eV, the peaks of C─O (≈286.5 eV), C═O (≈288.6.0 eV), and CF*_x_* (≈292.9 eV) bonds can be observed.^[^ ^61^, ^62^ ^]^ The low C─O and C═O content of the PT/DME‐FT electrode implies the formation of an inorganic‐rich SEI on the surface, which is known to be more favorable for Li^+^ transport compared to an organic‐rich SEI.^[^ ^61^ ^]^ The F 1s spectra in Figure 4b consist of two peaks at 685.0 and 687.6 eV, which are related to LiF and C─F (associated with TFSI^−^ and TTE) bonding, respectively.^[^ ^63^ ^]^ It is noted that both FEC and TTE additives were decomposed, releasing F^−^ ions to form LiF. FEC can be more favorably reduced,^[^ ^64^ ^]^ leading to a higher LiF content on the PT/DME‐F electrode compared to that on the PT/DME‐T electrode. This LiF‐rich SEI could be the root cause of the higher initial CE obtained for the former electrolyte.^[^ ^65^ ^]^ Adding FEC and TTE together seems to induce synergistic interaction, producing the highest LiF concentration (the mechanism is discussed later). Figure 4c shows the obtained N 1s spectra, in which a peak associated with pyrrolidinium cations (at ≈403.3 eV) and a peak associated with imide anions (at ≈399.5 eV) can be observed.^[^ ^55^ ^]^ In addition, a signal corresponding to Li_3_N (at ≈397.7 eV) appears. Similar to the trend of LiF, the PT/DME‐FT electrode shows the highest content of Li_3_N, followed by PT/DME‐F, PT/DME‐T, and PT/DME electrodes. Li_3_N, mainly derived from TFSI^−^, is more Li^+^ conductive than LiF. According to the literature,^[^ ^65^, ^66^ ^]^ the Li^+^ conductivities of LiF and Li_3_N are ≈10^−10^–10^−6^ and ≈10^−4^–10^−2^ S cm^−1^, respectively. Moreover, the Li_3_N‐rich SEI is protective, robust, and highly passivated.^[^ ^66^ ^]^ Figure 4d shows that the S 2p spectra can be deconvoluted into SO_2_^2−^ (≈168.1 eV), ‐NSO_2−_ (≈169.8 eV), and CF_3_SO_2−_ (≈171.0 eV) peaks. The first compound is associated with the decomposition product of TFSI^−^, whereas the latter two species are related to TFSI^−^ moieties.^[^ ^67^, ^68^ ^]^ The N 1s and S 2p data indicate that TFSI^−^ decomposition was greatly promoted in the PT/DME‐FT electrolyte. The B 1s spectra in Figure 4e exhibit a broad peak associated with B*_x_* O*_y_* species at ≈193.0 eV, which can be ascribed to DFOB^−^ decomposition. Again, the strongest peak intensity was found for the PT/DME‐FT electrode. The vigorous DFOB^−^ anion decomposition in this electrolyte also contributed to the predominant LiF signal in Figure 4b.

**Figure 5:**
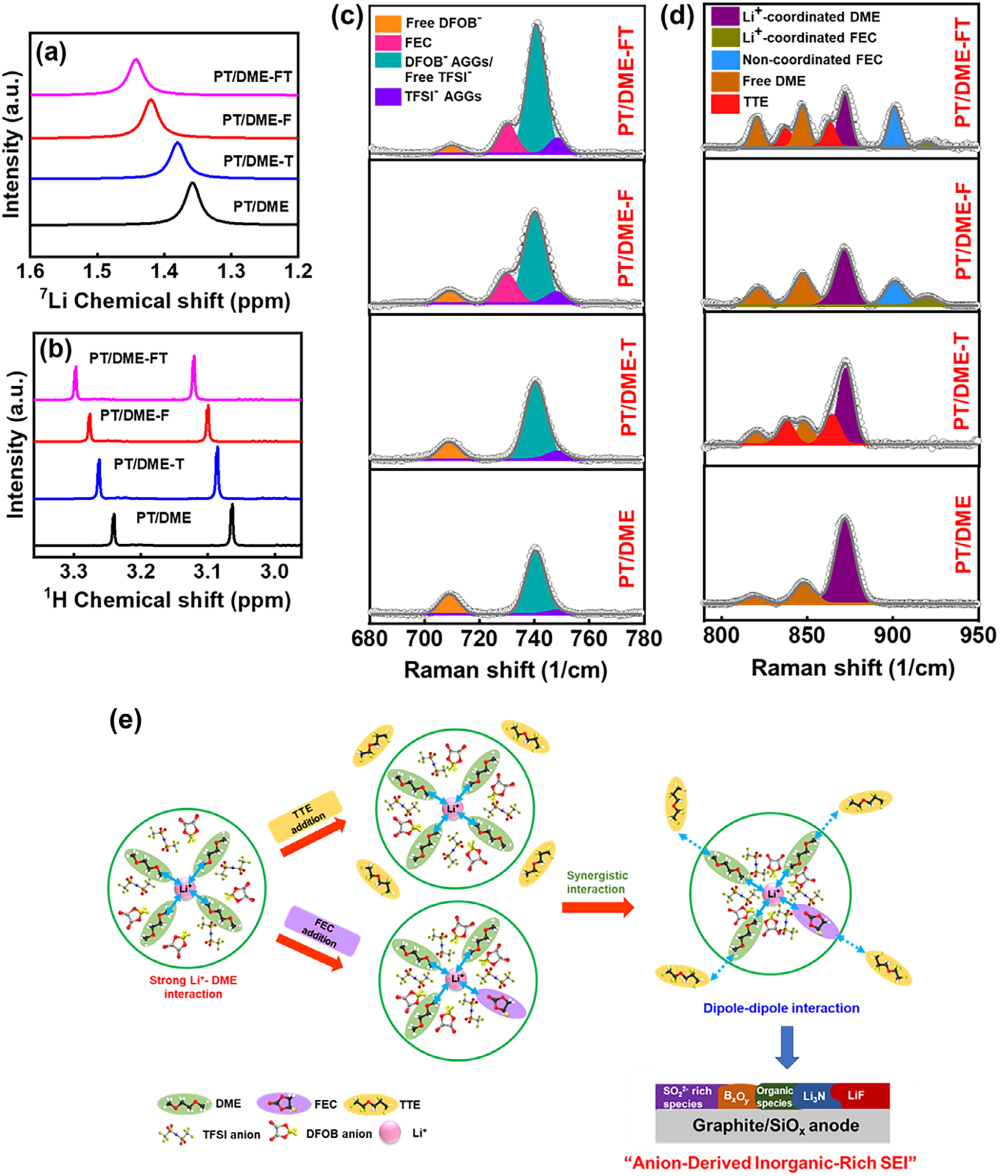
(a) ^7^Li and (b) ^1^H NMR spectra of various electrolytes. Raman spectra of various electrolytes in (c) 680−780 cm^−1^ and (d) 790−950 cm^−1^ regions. (e) Schematic illustration of Li^+^ coordination structures of various electrolytes and resulting SEI.

The Li^+^ solvation environments and solution structures of various electrolytes were investigated using solution nuclear magnetic resonance (NMR). **Figure** **5**a shows the ^7^Li NMR spectra obtained. The chemical shift of PT/DME‐FT is the largest (at 1.44 ppm), followed by PT/DME‐F (at 1.42 ppm) and PT/DME‐T (at 1.38 ppm); the baseline PT/DME electrolyte has the ^7^Li signal at 1.36 ppm. The clear downfield shift (toward a high ppm) due to the electrolyte additives indicates a deshielding effect of Li^+^ from the surrounding electron cloud, which suggests that the interaction between Li^+^ and the solvation sheath is weakened.^[^ ^69^, ^70^ ^]^ Figure 5b shows the ^1^H NMR data of various electrolytes. For the PT/DME electrolyte, two peaks appeared at ≈3.05 and ≈3.25 ppm, which are associated with the CH_3_ and CH_2_ groups, respectively, of DME.^[^ ^69^ ^]^ Compared to PT/DME, downfield shifts of the peaks were observed with the FEC and TTE additives. Notably, the strongest downfield shift was found for the PT/DME‐FT electrolyte. This suggests that the Li^+^−anion interaction was enhanced, which reduced the shielding effect of the anions on the DME solvent.^[^ ^71^, ^72^ ^]^

The solvation structure is also examined using Raman spectroscopy; the data are shown in Figure 5c,d. In the 680−780 cm^−1^ region,^[^ ^73^, ^74^ ^]^ the free DFOB^−^ and DFOB^−^ aggregates (AGGs) contribute to the characteristic peaks at 709 and 740 cm^−1^, respectively. The signals at 730 and 748 cm^−1^ are associated with FEC and TFSI^−^ AGGs, respectively. It is noted that the free TFSI‐peak overlaps at ≈740 cm^−1^. The addition of FEC outperforms that of TTE in reducing the free DFOB^−^ intensity. The PT/DME‐FT electrolyte exhibits the highest AGG peak intensity, whereas the free DFOB^−^ signal is minimal. This indicates that DFOB^−^ and TFSI^−^ anions are prone to coordinate with Li cations in the electrolyte. In the 790−950 cm^−1^ region,^[^ ^69^, ^75^ ^]^ two bands at 820 and 847 cm^−1^ correspond to free DME, while the band at 872 cm^−1^ is associated with Li^+^‐coordinated DME. The TTE bands are located at 837 and 862 cm^−1^. The peaks at 901 and 920 cm^−1^ are attributed to non‐coordinated FEC and Li^+^‐coordinated FEC, respectively. As shown, when the addition of TTE marginally affects the Li^+^ solvation structure, the FEC addition can clearly increase the free DME intensity and suppress the Li^+^‐coordinated DME signal. In the PT/DME‐FT electrolyte, the Raman intensity of both Li^+^‐coordinated FEC and Li^+^‐coordinated DME peaks are further reduced, confirming the enhanced Li^+^−anion interaction in the Li^+^ solvation sheath. The data in the two Raman regions are well‐aligned and consistent with the NMR results.

The solution structures of various electrolytes are shown in Figure 5e. The weakly solvating TTE molecules alone can barely engage in the Li^+^ solvation sheath. The function of TTE is to induce a locally concentrated Li salt and marginally enhance the Li^+^−anion interaction. FEC partially replaces DME in the first Li^+^ solvation sheath.^[^ ^76^ ^]^ Thus, the Li^+^‐coordinated FEC contributes to early SEI formation and surface passivation.^[^ ^64^ ^]^ Because the solvation power of FEC is lower than that of DME, a clear deshielding of Li^+^ (Figure 5a) was observed. An intriguing variation of the Li^+^ coordination structure was found when FEC and TTE additives were simultaneously applied. A synergistic effect can be observed. As shown in the scheme, the Li^+^ is surrounded by a high‐entropy environment, consisting of DME, FEC, TTE, TFSI^−^, DFOB^−^, and PMP^+^. The inter‐molecular dipole‐dipole interaction between TTE and polar FEC molecules can drive the latter apart from Li^+^,^[^ ^77^ ^]^ increasing the proportion of TFSI^−^ and DFOB^−^ anions in the Li^+^ solvation sheath. Moreover, the participation of the anions in the Li^+^ solvation sheath and the dipole dipole interaction between TTE and DME^[^ ^69^ ^]^ further weaken the solvation energy between Li^+^ and DME. This argument is supported by the NMR and Raman data, which showed the largest shifts for the PT/DME‐FT electrolyte. Thus, the Li^+^ ions drive the anions toward the anode, leading to the formation of a LiF‐, Li_3_N‐, B*_x_* O*_y_*‐, and SO_2_^2−^‐rich SEI (as illustrated in Figure 5e). This anion‐derived inorganic‐rich SEI provides good Li^+^ transport kinetics and high stability.^[^ ^78^ ^]^ Furthermore, the weakened Li^+^/DME interaction facilitates the Li^+^ desolvation process and prevents the DME from co‐intercalation. As a consequence, the graphite anode has great rate capability and cycling stability. Of note, the presence of high‐concentration PMP^+^ cations in the high‐entropy electrolyte could be crucial, since they may compete with Li^+^ to coordinate with solvents, leading to a reduced size of Li^+^ solvation clusters and decreased Li^+^ solvation energy.

We also tested the compatibility of the developed PT/DME‐FT electrolyte for a SiO*_x_* anode. Figure S9a,b (Supporting Information) shows the charge–discharge curves of the SiO*_x_* electrodes measured in the conventional 1 M LiPF_6_ EC/DEC electrolyte and the PT/DME‐FT electrolyte, respectively. Regardless of the current rate (0.2 to 5 A g^−1^), the reversible capacities obtained in the latter electrolyte are clearly higher than those obtained in the former electrolyte. Figure S9c (Supporting Information) compares the cycling stability data measured in the two electrolytes at 0.5 A g^−1^. After 200 charge–discharge cycles, the electrodes retained 69% and 88% of their initial capacities, respectively. The experimental results indicate that the proposed PT/DME‐FT electrolyte is also applicable to SiO*_x_*‐based anodes for improving electrochemical performance.

**Figure 6:**
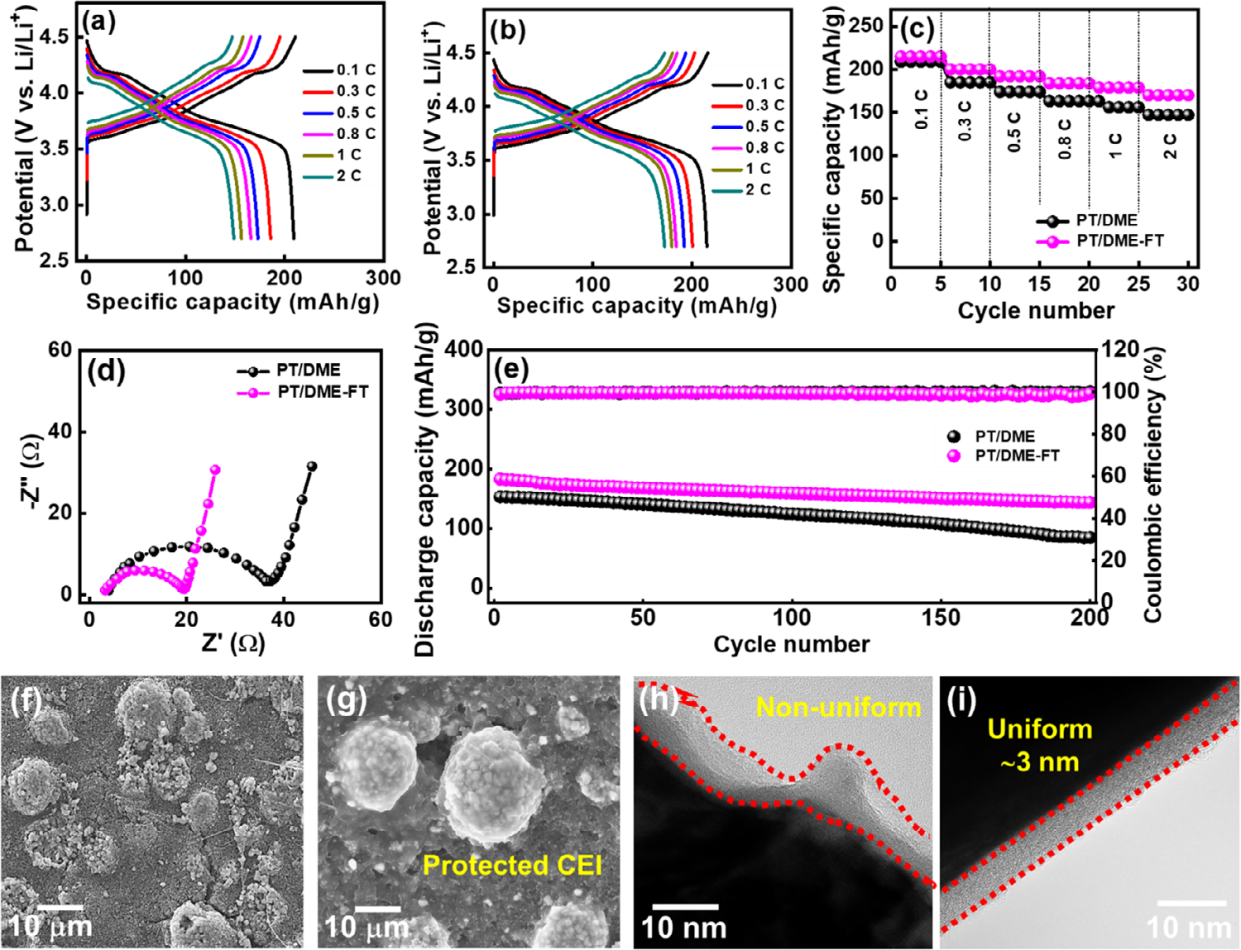
Charge‐discharge curves of NMC‐811 cathodes measured at various rates in (a) PT/DME and (b) PT/DME‐FT electrolytes. (c) Comparative rate performance, (d) EIS spectra, and (e) cycling stability data (at 1 C) of NMC‐811 cathodes with various electrolytes. SEM and TEM images of NMC‐811 electrodes after being tested in (f,h) PT/DME and (g,i) PT/DME‐FT electrolytes for 200 cycles.

The electrolyte effects on the cathode part were examined. **Figure** **6**a,b shows the charge–discharge profiles of the NMC‐811 cathodes recorded with an upper cutoff voltage of 4.5 V. The reversible capacities at 0.1 C are 209 and 215 mAh g^−1^ for the PT/DME and PT/DME‐FT cells, respectively. Figure 6c shows that the measured capacities decrease to 147 and 172 mAh g^−1^ at 2 C, respectively, corresponding to 70% and 80% of the capacities measured at 0.1 C (**Table** **3**). To examine the impedance of various cells, EIS measurements were conducted. The obtained Nyquist data in Figure 6d show that the *R*_ct_ values for the PT/DME and PT/DME‐FT cells are 36.3 and 19.4 Ω, respectively. The synergy of FEC and TTE results in a low *R*_ct_, leading to the superior rate capability of the NMC‐811 electrode. Figure 6e shows the cyclability of NMC‐811 cathodes with PT/DME and PT/DME‐FT electrolytes. After 200 cycles, the capacity retention ratios are 57 and 80%, respectively. We confirm that the proposed PT/DME‐FT electrolyte clearly outperformed the conventional LiPF_6_ EC/DEC electrolyte (see Figure S10, Supporting Information) in terms of the NMC‐811 rate capability and cycling stability (145 mAh g^−1^ at 2 C and 50% capacity retention after 200 cycles for the latter electrolyte). The PT/DME‐FT electrolyte is highly compatible not only with the graphite anode but also with the high‐Ni NMC‐811 cathode up to 4.5 V. This finding is in line with the hypothesis, proposed by Manthiram et al., that the electrolyte composition is crucial.^[^ ^44^ ^]^ The PT/DME‐FT electrolyte can mitigate the surface reactivity of NMC‐811 and stabilize the electrode charge–discharge performance upon cycling.

Figure 6f,g shows the postmortem SEM images of the NMC‐811 electrodes after cycling. The morphology of the electrode in the PT/DME electrolyte was clearly distorted after cycling. Loosened particles and surface cracks were found. Of note, using the PT/DME‐FT electrolyte well maintained the electrode structural integrity. A distinct protection layer covering the electrode surface was observed, which is thought to be related to the cathode‐electrolyte interphase (CEI) derived from the FEC and TTE additives that can prevent the NMC‐811 electrode from substantially deteriorating. Figure 6h,i shows the postmortem TEM images of the NMC‐811 particles after 200 charge–discharge cycles. A non‐uniform and relatively thick CEI layer was found for the PT/DME sample. Presumably, the repeated breakdown and growth of the infirm CEI made it accumulate upon cycling, leading to a rough interface layer. In contrast, a compact, continuous, and uniform CEI with a thickness of ∼3 nm formed in the PT/DME‐FT electrolyte. This conformal and well‐adhered protective layer is considered crucial for the great cyclability of the electrode.

In addition, we assessed the transition metal dissolution properties of the NMC‐811 electrodes upon cycling in 1 M LiPF_6_ EC/DEC and PT/DME‐FT electrolytes at 50 °C. After 100 charge–discharge cycles, the separators were extracted from the cells and subjected to energy‐dispersive X‐ray spectroscopy analysis. The obtained data are shown in Figure S11 (Supporting Information). With the conventional carbonate electrolyte, the observed Mn and Ni peak intensities are clearly higher than those found for the PT/DME‐FT electrolyte. The high voltage of 4.5 V and the produced HF (from the conventional LiPF_6_ electrolyte) can corrode NMC‐811, leading to metal dissolution.^[^ ^79^ ^]^ In contrast, the PT/DME‐FT electrolyte can withstand high voltage (see Figure 1). Moreover, a unique protective CEI layer forms, suppressing the attack from the electrolyte (see Figure 6). As a result, the metal dissolution side reactions decreased, leading to the superior cyclability of the NMC‐811 electrode.

Compared to the reported LiFSI PMP–FSI/TTE electrolyte,^[^ ^36^ ^]^ which has been the only IL‐ether composite electrolyte for carbonaceous anodes of LIBs, the PT/DME‐FT electrolyte possesses higher ionic conductivity (6.3 vs 3.2 mS cm^−1^) and enable better graphite cyclability (82 vs 76% capacity retention after 300 cycles). Of note, limited by the high corrosivity of FSI^−^,^[^ ^37^ ^]^ the voltage window and cathode compatibility of LiFSI PMP–FSI/TTE electrolyte were not reported. In contrast, the proposed PT/DME‐FT was confirmed to have great high‐voltage stability.

**Figure 7:**
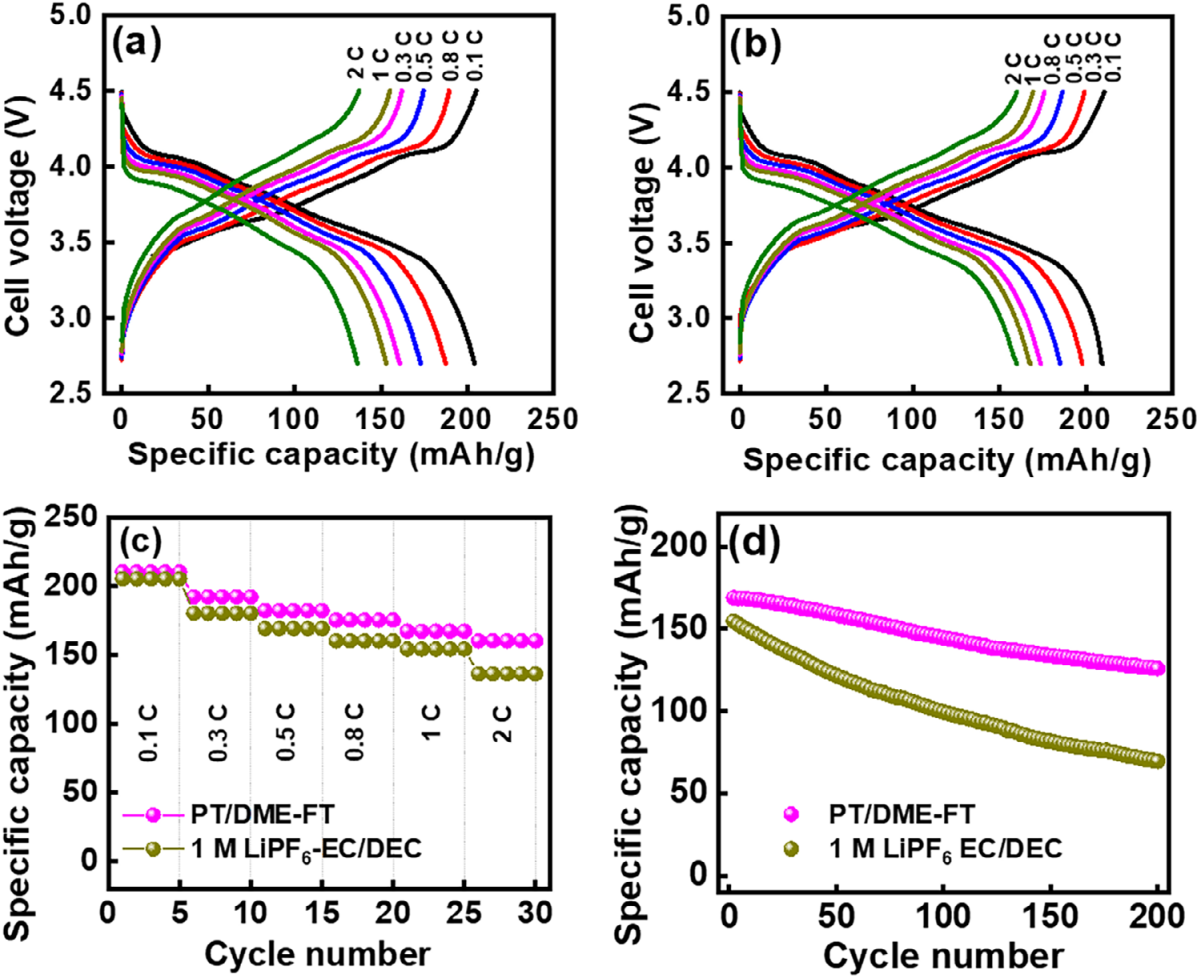
Charge‐discharge profiles of graphite||NMC‐811 full cells with (a) 1 M LiPF_6_ EC/DEC and (b) PT/DME‐FT electrolytes measured at various rates. (c) Comparative rate performance and (d) cycling stability data (at 1 C) of graphite||NMC‐811 full cells with two electrolytes.

To evaluate the potential of the proposed high‐entropy electrolyte for practical applications, a graphite||NMC‐811 full cell was constructed with an anode‐to‐cathode capacity ratio of 1.1 and operated at a cell voltage of up to 4.5 V. **Figure** **7**a,b shows the charge‐discharge profiles measured at various C rates (1 C = 200 mA g^−1^ for NMC‐811) for 1 m LiPF_6_ EC/DEC and PT/DME‐FT cells, respectively. As compared in Figure 7c, the reversible specific capacities (based on the cathode mass) of the latter cell are higher than those of the former cell, especially at high rates. For instance, at 2 C, the measured specific capacities are 136 and 160 mAh g^−1^, respectively, corresponding to 66% and 76% of the capacity values measured at 0.1 C. Figure 7d shows the cycling stability data of the two cells measured at 1 C. After 200 cycles, the conventional and PT/DME‐FT cells retained 46% and 75% of their initial capacities, respectively. The cycling stability of the two cells was also evaluated at 50 °C; the obtained data are shown in Figure S12 (Supporting Information). The capacity retentions after 100 cycles were 35% and 68%, respectively, indicating the superior durability of the PT/DME‐FT cell at elevated temperatures. This promising performance of the proposed PT/DME‐FT electrolyte can be attributed to its great compatibility with the graphite anode and Ni‐rich NMC‐811 cathode and the reliable electrode/electrolyte interface layers generated during high‐voltage charging‐discharging.

## 3 Conclusion

An electrolyte composed of Li^+^, PMP^+^, TFSI^−^, DFOB^−^, DME, FEC, and TTE was proposed. Without using a high concentration of Li salt, this IL‐based high‐entropy electrolyte has several desirable characteristics, such as low flammability, high thermal stability, negligible corrosivity, and a large potential window. This electrolyte also solves the high viscosity and low conductivity problems of regular IL electrolytes. In addition, the long‐standing issue of PMP^+^ and DME co‐intercalation into the graphite lattice is eliminated, as verified in our operando XRD study. The substantial ^7^Li NMR peak shifts indicate a strong deshielding effect of Li^+^, which suggests that the interaction between Li^+^ and solvent molecules is weakened, and thus, the anions have a higher chance to participate in the coordination sheath in this high‐entropy PT/DME‐FT electrolyte. This unique Li^+^ coordination structure, confirmed by NMR and Raman analyses, leads to a facile Li^+^ desolvation process and the formation of a LiF‐, Li_3_N‐, B*_x_* O*_y_*‐, and SO_2_^2−^‐rich robust SEI. As a result, the graphite and SiO*_x_* anodes had great rate capability and cycling stability. Moreover, the proposed electrolyte is highly compatible with a high‐Ni NCM‐811 cathode, forming a good CEI to stabilize the electrode surface and minimize transition metal dissolution. A high‐performance 4.5‐V LiNi_0.8_Co_0.1_Mn_0.1_O_2_//graphite full cell was validated. The results indicate that the proposed high‐entropy electrolyte is promising for high‐energy‐density and high‐safety LIB applications.

## 4 Experimental Section

### Electrolyte Preparation

PMP−TFSI IL (purity: 99.9%), purchased from Solvionic Co., Ltd., was vacuum‐dried at 80 °C for 24 h before use. Battery‐grade LiFSI and LiPF_6_ were purchased from Kishida Chemical Co., Ltd. LiDFOB and LiTFSI were purchased from Solvionic Co., Ltd. Battery‐grade DME from Kishida Chemical Co., Ltd. was blended with PMP–TFSI IL at a 1:1 volume ratio with 1 m Li salt to form a composite electrolyte. 5 wt.% of FEC, TTE, or FEC/TTE (i.e., 2.5 wt.% each) was used as the electrolyte additive. A conventional electrolyte, consisting of 1 m LiPF_6_ and EC/DEC (1:1 by volume) mixed solvent, was used for comparison. All the electrolytes were prepared in an Ar‐filled glove box and dried over fresh molecular sieves for 8 h before use. The electrolyte water content, measured using a Karl Fisher titrator, was typically below 25 ppm. The ionic conductivity and viscosity of the electrolytes were examined using a TetraCon 325 conductivity meter and a Brookfield DV‐I viscometer, respectively.

### Electrode Preparation and Cell Assembly

Graphite anode powder (MG11 type) was provided by China Steel Chemical Corporation. NMC‐811 cathode powder was provided by Amita Technologies Inc. The electrode slurry was prepared by mixing the active material, Super P, and polyvinylidene fluoride binder in a weight ratio of 80:10:10 in *N*‐methyl‐*2*‐pyrrolidone solvent. The slurry was cast onto an Al or Cu current collector using a doctor blade and then vacuum‐dried at 100 °C for 12 h. The graphite and NMC‐811 mass loading on the electrodes were ≈3.1 and ≈5.0 mg cm^−2^, respectively. The obtained electrodes were roll‐pressed and punched to match the required dimensions of a CR2032 coin cell. Li foil and a glass fiber membrane were used as the counter electrode and separator, respectively. The cell assembly was done in an Ar‐filled glove box (Vigor Tech. Co. Ltd.), where both the water and oxygen content levels were below 0.2 ppm.

### Material and Electrochemical Characterizations

The morphologies and microstructures of the samples were analyzed using SEM (FEI Inspect F50) and TEM (FEI Tecnai F20). The crystallinity was examined using XRD (Bruker D2 PHASER). TGA (Perkin–Elmer TGA7) was conducted to evaluate the thermal stability of the electrolytes. X‐ray photoelectron spectroscopy (Thermo Fisher Scientific ESCALAB 250Xi) was used to analyze the electrode surface chemical composition. Monochromatic Al K_α_ radiation (1486.6 eV) was adopted as the X‐ray source. The Raman spectra of various electrolytes were recorded with a UniDRON Raman spectrophotometer (the laser wavelength is 532 nm). The ^1^H NMR and ^7^Li NMR spectra of various electrolytes were measured with a JEOL ECZ500R/S1 spectrometer using CDCl_3_ as a solvent. The electrolyte flammability was tested following the procedures suggested in a previous paper.^[^ ^80^ ^]^ Briefly, the electrolyte was adsorbed in a glass fiber membrane and then tested with an electric Bunsen burner under air. The distance between the electrolyte and the flame was ≈12 cm. Linear sweep voltammetry was carried out using a Biologic VSP‐300 potentiostat. The galvanostatic charge–discharge properties (such as capacity, rate capability, and cycling stability) of the cells was evaluated using a NEWARE CT‐4000 battery tester at 25 °C. The voltage ranges used for the graphite and NCM‐811 half cells were 0.01–1.5 V and 2.7–4.5 V, respectively. For the operando XRD study, the cells were cycled at a rate of 0.1 C. EIS measurements were carried out with a Biologic VSP‐300 potentiostat in a frequency range of 10^6^–10^−2^ Hz with a voltage perturbation amplitude of 10 mV. To construct graphite||NCM‐811 full cells with various electrolytes, an anode‐to‐cathode capacity ratio of ≈1.1 was used.

### Statistical Analysis

For the EIS and charge‐discharge measurements, at least five duplicate cells were repeated to ensure the validity. The data deviation was typically within ≈3% and the reported values were the medians. All the XPS spectra were calibrated with the binding energy of C 1s at 284.6 eV. The data fitting was conducted using XPSPEAK 4.1 software. The Origin software was used for data analysis and processing.

## Conflict of Interest

The authors declare no conflict of interest.

## Supporting information



Supporting Information

## Data Availability

The data that support the findings of this study are available from the corresponding author upon reasonable request.
