# Supplementary material for: High‐Entropy Non‐Flammable Ionic Liquid/Dimethoxymethane Composite Electrolyte for High‐Performance Lithium‐Ion Batteries
Source: Adv Sci (Weinh). 2025 Mar 17;12(18):2417306. doi: 10.1002/advs.202417306 (PMC12079477; doi:10.1002/advs.202417306)
Supplement: Supplementary file 1 — Supporting Information [file ADVS-12-2417306-s001.docx]

Copyright WILEY-VCH Verlag GmbH & Co. KGaA, 69469 Weinheim, Germany, 2024.

Supporting Information

**High-Entropy Non-Flammable Ionic Liquid/Dimethoxymethane Composite Electrolyte for High-Performance Lithium-Ion Batteries**

Purna Chandra Rath^§^, Chun-Yen Chen^§^, Jagabandhu Patra, Chun-Chen Yang, Yu-Sheng Su, Chien-Te Hsieh, Wei-Ren Liu, Ju Li, Jeng-Kuei Chang*


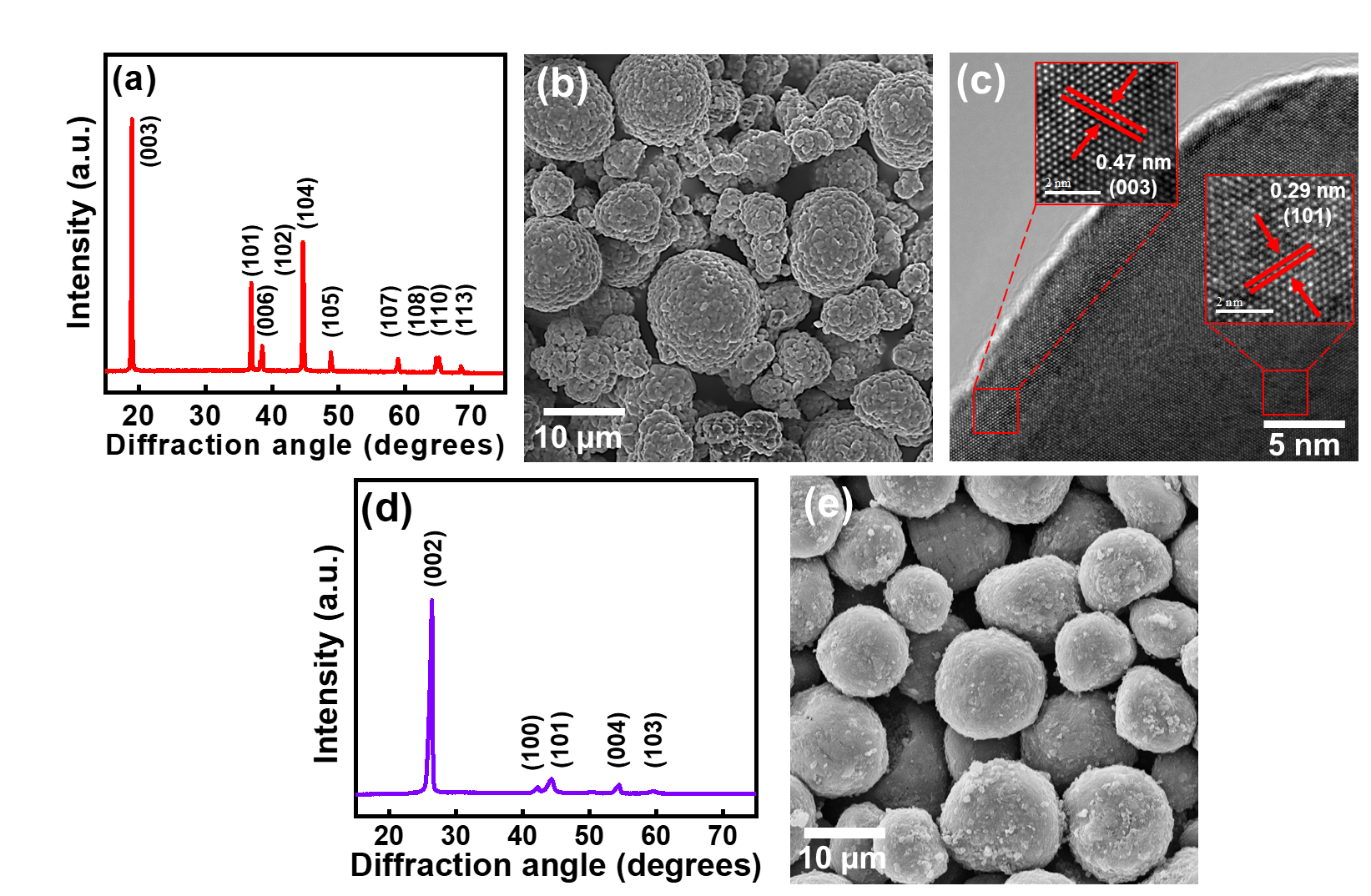


**Figure S1.** (a) XRD pattern, (b) SEM image, (c) high-resolution TEM images of NMC-811 sample. (d) XRD pattern and (e) SEM image of graphite sample.


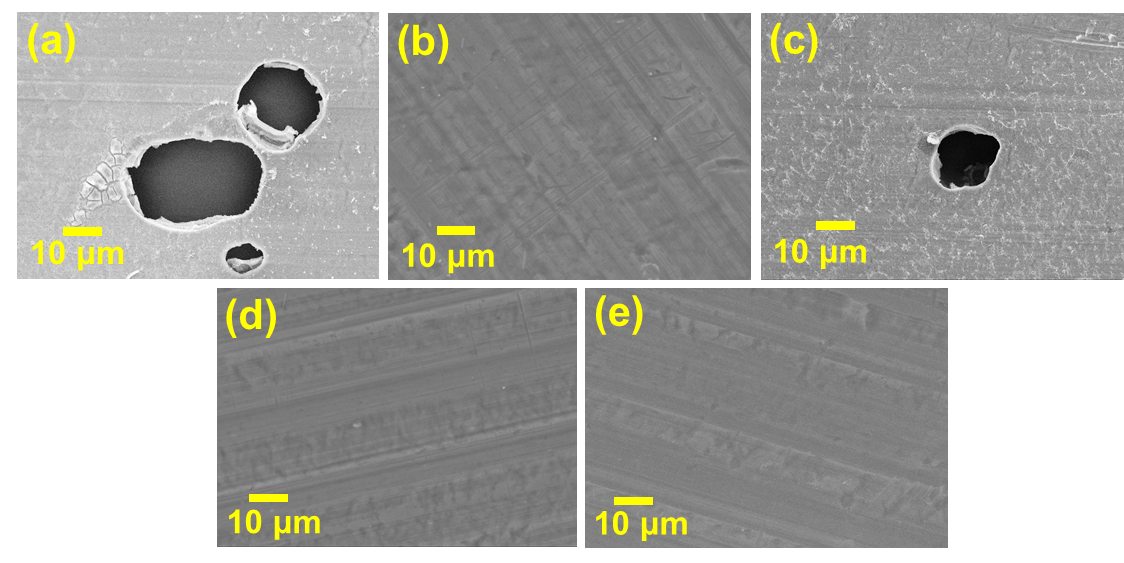


**Figure S2.** SEM image of Al electrodes after linear sweep voltammetry tests in (a) 1 M LiFSI PMP−TFSI/DME, (b) 1 M LiPF_6_ PMP−TFSI/DME, (c) 1 M LiTFSI PMP−TFSI/DME, (d) 1 M LiDFOB PMP−TFSI/DME, and (e) 1 M LiPF_6_ EC/DEC electrolytes.


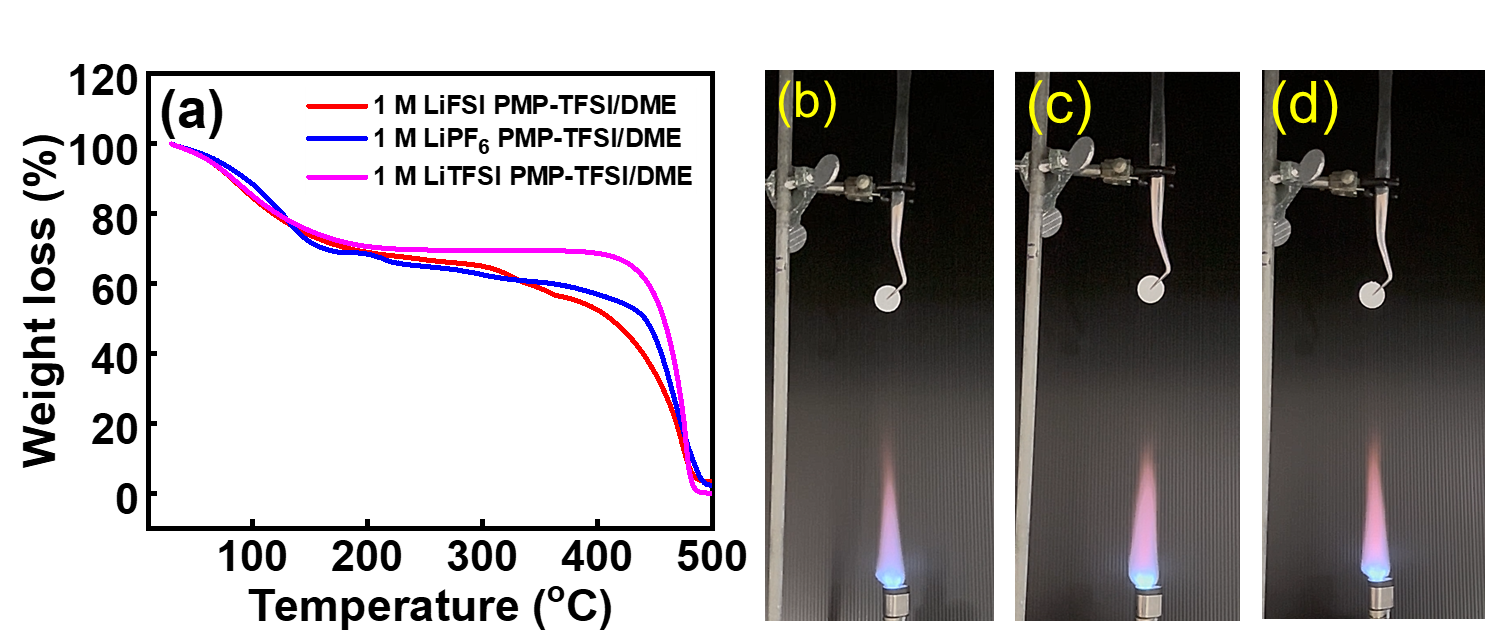


**Figure S3.** (a) TGA data of PMP−TFSI/DME electrolytes with various salts. Flammability tests of (b) 1 M LiFSI PMP−TFSI/DME, (c) 1 M LiPF_6_ PMP−TFSI/DME, and (d) 1 M LiTFSI PMP−TFSI/DME electrolytes.


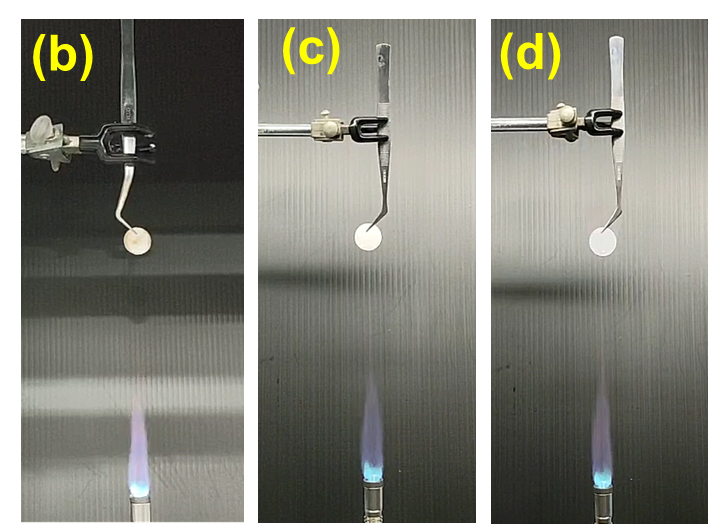


**Figure S4.** (a) TGA data of PT/DME electrolytes with various additives. Flammability data of (b) PT/DME-F, (c) PT/DME-T, and (d) PT/DME-FT electrolytes.

**Figure S5.** Electrochemical stability windows of PT/DME, PT/DME-F, PT/DME-T, and PT/DME-FT electrolytes recorded at Al electrodes.

**Figure S6.** (a) Initial charge-discharge profiles measured at 0.1 C and (b) charge-discharge curves measured at various rates for graphite anode in 1 M LiPF_6_ EC/DEC electrolyte.

**Figure S7.** Charge-discharge curves of graphite anodes measured at various rates in (a) PT/DME, (b) PT/DME-F, and (c) PT/DME-T electrolytes.

**Figure S8.** (a) EIS spectra obtained after 300 cycles and (b) variation of *R*_ct_ of graphite anodes in various electrolytes after cycling. SEM images of graphite anodes after being tested in (c) PT/DME and (d) PT/DME-FT electrolytes for 300 cycles
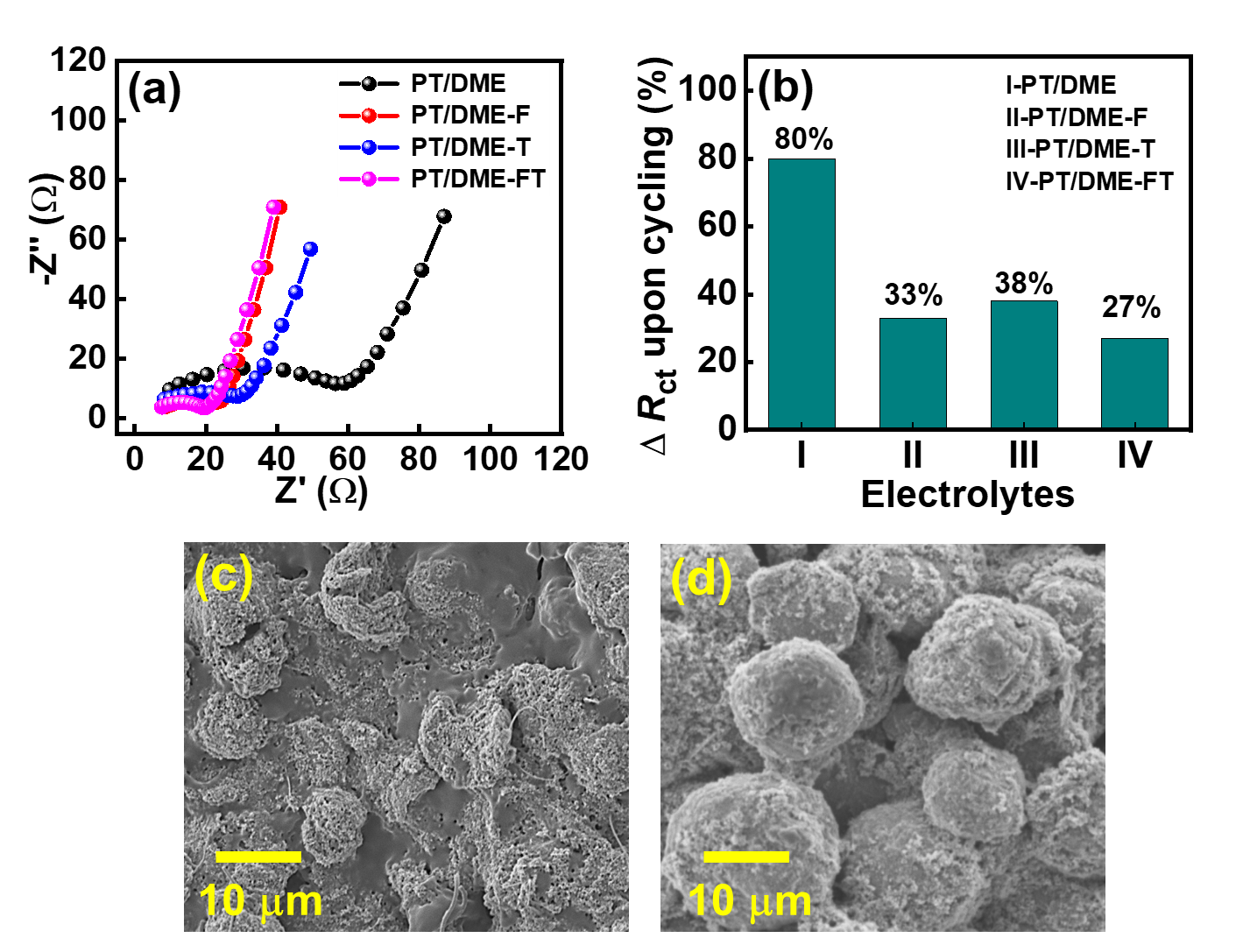
.


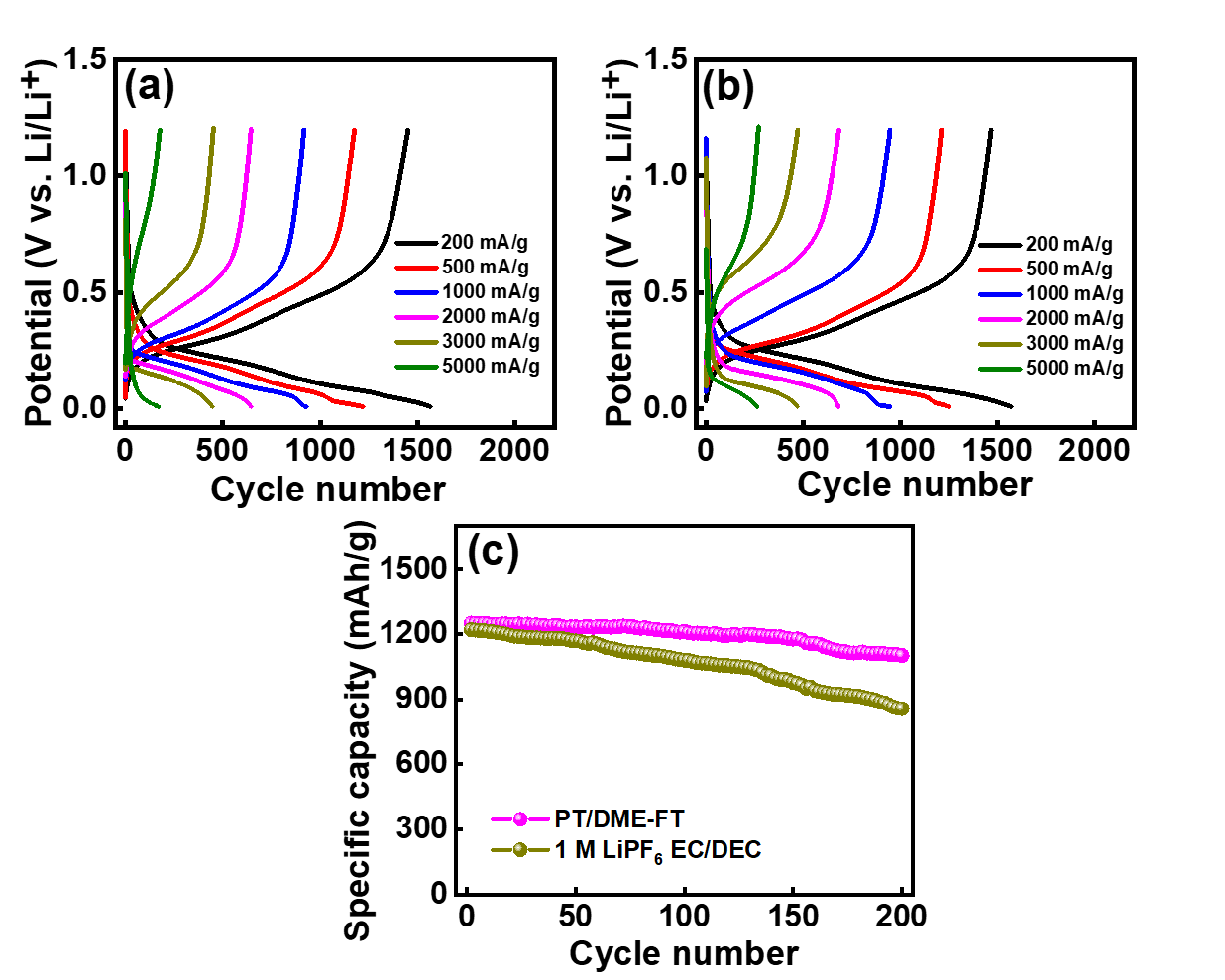


**Figure S9.** Charge-discharge curves of SiO*_x_* anodes measured at various rates in (a) 1 M LiPF_6_ EC/DEC and (b) PT/DME-FT electrolytes. (c) Cycling stability data of SiO*_x_* anodes measured at 0.5 A g^−1^ with two electrolytes for 200 cycles.


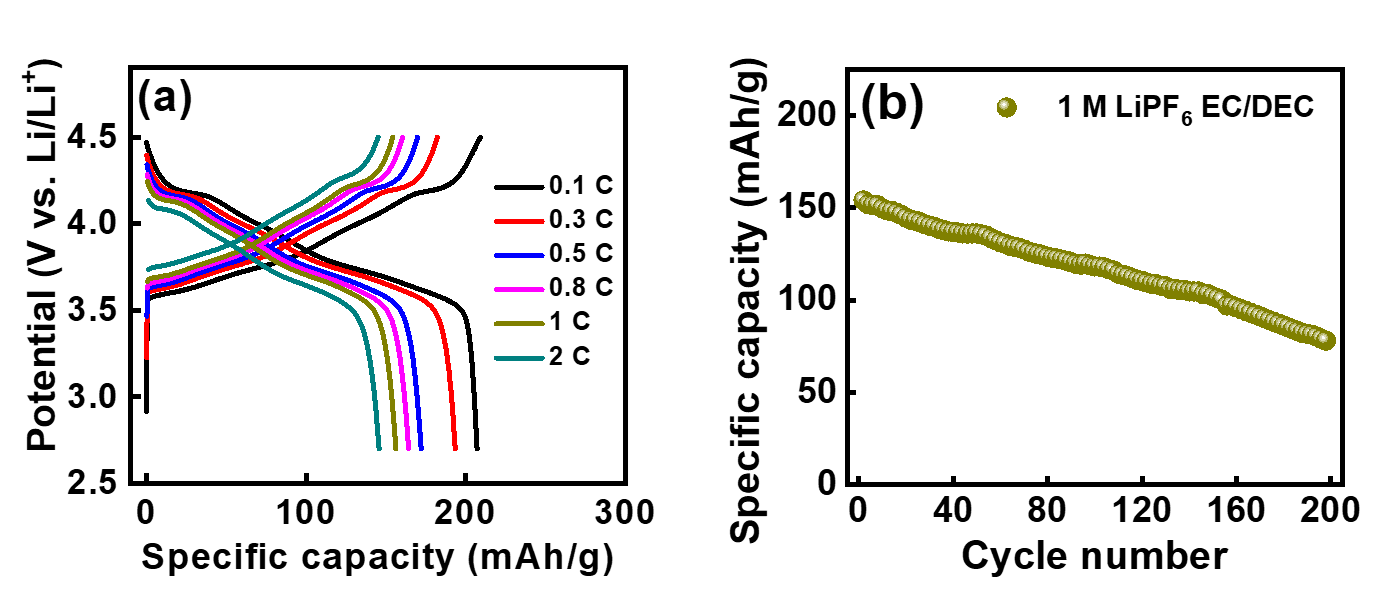


**Figure S10.** (a) Charge-discharge curves measured at various rates and (b) cycling stability data measured at 1 C for NMC-811 cathode in 1 M LiPF_6_ EC/DEC electrolyte.


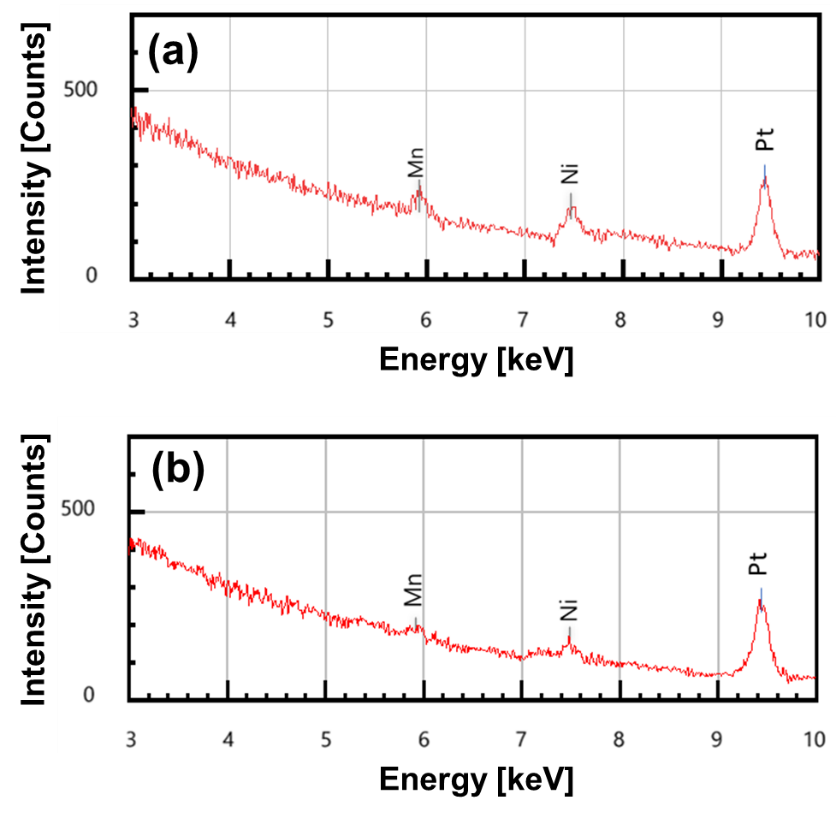


**Figure S11.** Energy-dispersive X-ray spectroscopy analyses on separators extracted from NMC-811 cells with (a) 1 M LiPF_6_ EC/DEC and (b) PT/DME-FT electrolytes after 200 charge-discharge cycles at 50 °C.

**Figure S12.** Cycling stability data of graphite||NMC-811 full cells (measured at @ 1 C) with two electrolytes measured at 50 °C.
